# Rapid evolution of HIV-1 to functional CD8+ T-cell rResponses in humanized BLT mice

**DOI:** 10.1186/1742-4690-9-S2-P249

**Published:** 2012-09-13

**Authors:** TE Dudek, DC No, E Seung, V Vrbanac, L Fadda, KF Bryant, M Altfeld, AD Luster, DM Knipe, AM Tager, TM Allen

**Affiliations:** 1 Ragon Institute of MGH, MIT and Harvard, Charlestown, MA, USA; 2 Center for Immunology and Inflammatory Diseases, MGH, Boston, MA, USA; 3 Harvard Medical School, Boston, MA, USA

## Background

The newly developed humanized BLT (bone marrow, liver, thymus) mouse model holds great promise to facilitate the in vivo study of human immune responses. However, little data exists regarding the extent to which cellular immune responses in humanized BLT mice accurately reflect those seen in humans.

## Methods

Multiple sets of humanized BLT mice reconstituted with distinct human tissues were infected with the HIV-1 molecular clone JR-CSF. Mice were then bled every two weeks to measure plasma viral loads, sequence evolution and cellular immune responses.

## Results

During the acute phase of infection BLT mice rapidly mounted multiple HIV-1-specific CD8+ cellular immune responses against normally immunodominant human CD8 epitopes, with rapid and reproducible viral escape observed within epitopes that similarly tend to escape early in humans. CD8+ T cell responses and viral escape to these same epitopes was confirmed in mice reconstituted with distinct human tissue but expressing the same restricting HLA alleles. Importantly, in two independent groups of mice expressing HLA-B*57 we observed the rapid induction of CD8+ T-cell responses against a number of B*57-restricted responses at frequencies similar to those seen in humans, including the normally immunodominant B*57-IW9, -KF11 and -TW10 epitopes in Gag from which the virus failed to rapidly escape. As in humans, the presence of these conserved responses correlated with significantly greater control over early viral replication. Preliminary vaccine studies in BLT mice support the ability of conventional approaches to induce CD8+ T cell responses and suppress viral loads.

## Conclusion

These studies indicate that the specificity, magnitude, and immunodominance patterns of human CD8+ T-cell responses in humanized BLT mice appear to closely reflect those of humans. These data support the potential of humanized BLT mice to significantly advance HIV-1 vaccine development, providing a critical new tool to complement the SIV infected macaque model.

